# Characteristics of immune and inflammatory responses among different age groups of pediatric patients with COVID-19 in China

**DOI:** 10.1007/s12519-021-00440-1

**Published:** 2021-08-02

**Authors:** Su-Qiong Ji, Min Zhang, Yong Zhang, Kun Xia, Yuan Chen, Qian Chu, Yong-Chang Wei, Fu-Ling Zhou, Bi-Tao Bu, Hong-Lei Tu, Ya-Yun Cao, Li-Ya Hu

**Affiliations:** 1 Department of Neurology, Tongji Hospital, Tongji Medical College, Huazhong University of Science and Technology, Wuhan, China; 2 Department of Cardiovascular Medicine, Wuhan Children’ Hospital, Tongji Medical College, Huazhong University of Science and Technology, Wuhan, China; 3 Department of Geriatrics, Tongji Hospital, Tongji Medical College, Huazhong University of Science and Technology, Wuhan, China; 4 Department of Oncology, Tongji Hospital, Tongji Medical College, Huazhong University of Science and Technology, Wuhan, China; 5 Department of Radiation and Medical Oncology, Hubei Key Laboratory of Tumor Biological Behaviors, Hubei Cancer Clinical Study Center, Zhongnan Hospital of Wuhan University, Wuhan, China; 6 Department of Hematology, Zhongnan Hospital of Wuhan University, Wuhan, China

**Keywords:** Age difference, China, Coronavirus disease 2019, Immune, Inflammation, Pediatric

## Abstract

**Background:**

Severe cases of coronavirus disease 2019 (COVID-19) among pediatric patients are more common in children less than 1 year of age. Our aim is to address the underlying role of immunity and inflammation conditions among different age groups of pediatric patients.

**Methods:**

We recruited pediatric patients confirmed of moderate COVID-19 symptoms, admitted to Wuhan Children's Hospital from January 28th to April 1st in 2020. Patients were divided into four age groups (≤ 1, 1–6, 7–10, and 11–15 years). Demographic information, clinical characteristics, laboratory results of lymphocyte subsets test, immune and inflammation related markers were all evaluated.

**Results:**

Analysis included 217/241 (90.0%) of patients with moderate clinical stage disease. Average recovery time of children more than 6 years old was significantly shorter than of children younger than 6 years (*P* = 0.001). Reduced neutrophils and increased lymphocytes were significantly most observed among patients under 1 year old (*P* < 0.01). CD19+ B cells were the only significantly elevated immune cells, especially among patients under 1 year old (cell proportion: *n* = 12, 30.0%, *P* < 0.001; cell count: *n* = 13, 32.5%, *P* < 0.001). While, low levels of immune related makers, such as immunoglobulin (Ig) G (*P* < 0.001), IgA (*P* < 0.001), IgM (*P* < 0.001) and serum complement C3c (*P* < 0.001), were also mostly found among patients under 1 year old, together with elevated levels of inflammation related markers, such as tumor necrosis factor γ (*P* = 0.007), interleukin (IL)-10 (*P* = 0.011), IL-6 (*P* = 0.008), lactate dehydrogenase (*P* < 0.001), and procalcitonin (*P* = 0.007).

**Conclusion:**

The higher rate of severe cases and long course of COVID-19 among children under 1 year old may be due to the lower production of antibodies and serum complements of in this age group.

**Supplementary Information:**

The online version contains supplementary material available at 10.1007/s12519-021-00440-1.

## Introduction

The outbreak of coronavirus disease 2019 (COVID-19) caused by severe acute respiratory syndrome coronavirus 2 (SARS-CoV-2) infection has now spread worldwide [1] among nearly 200 countries [2]. Although people of all ages are susceptible to SARS-CoV-2 infection [3, 4], studies have shown distinctive differences between pediatric patients and adult patients [5]. Confirmed cases among children usually have relatively mild symptoms [5, 6]. The number of severe cases and mortality rate were significantly low [7] in pediatric patients. Immune and inflammatory conditions are proved to be the underlying reasons and influential factors. Immune status may even vary among children of different age groups [8, 9]. The largest retrospective pediatric clinical study of COVID-19 from Dong et al.’s study [4] has identified that the severe rate was especially high among children under 1 year old (severe cases: < 1 year, *n* = 33, 29.5%; critical cases: < 1 year, *n* = 7, 53.8%) compared with other age groups of pediatric patients. Whereas, relevant reports related to immune and inflammatory response among different age groups of pediatric patients are still rare.

**Fig. 1:**
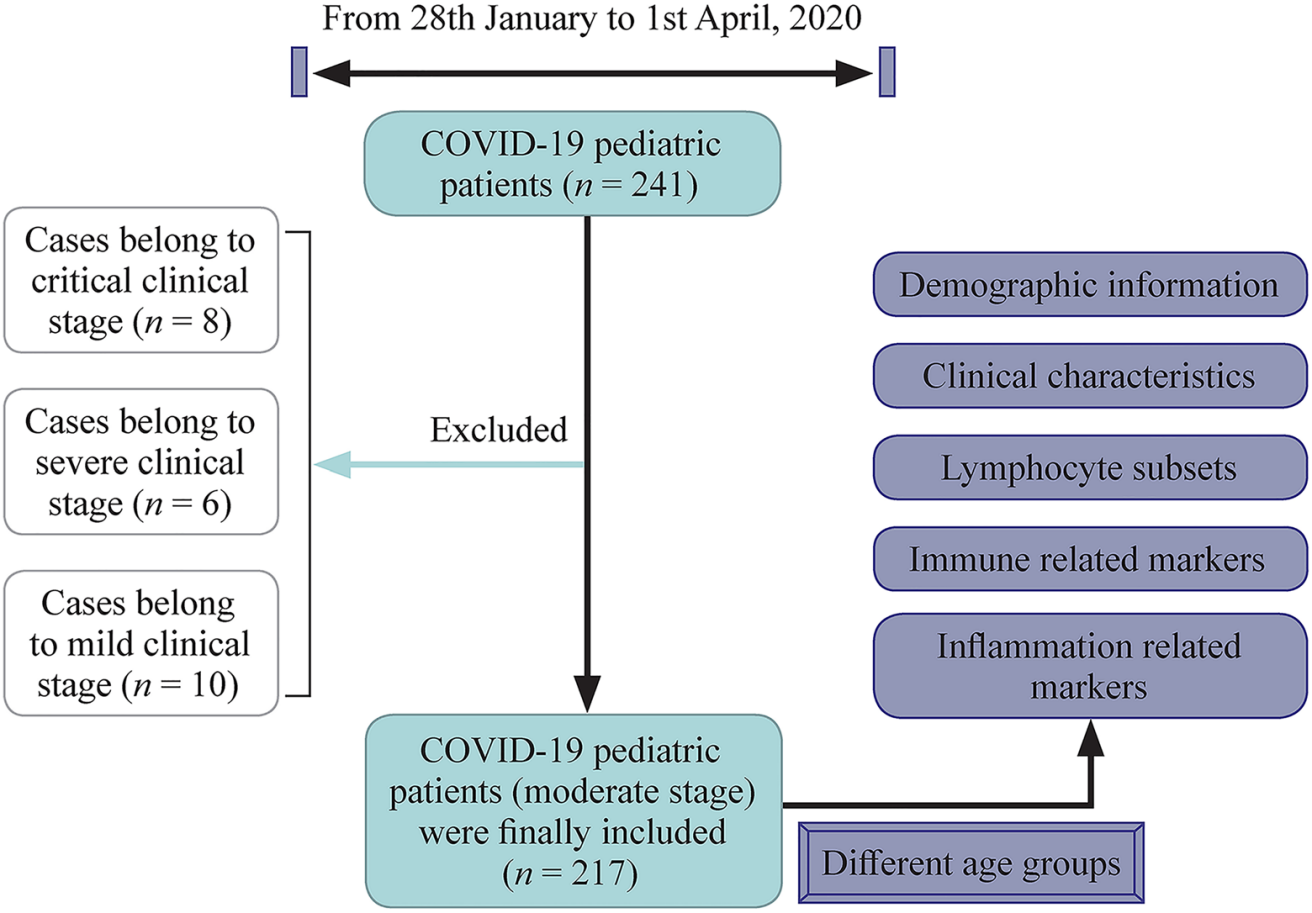
Scheme of the study.* COVID-19* coronavirus disease 2019

To better understand the immune and inflammatory responses, we aimed to comprehensively compare the levels of lymphocyte subsets, immune related components, inflammatory cytokines, and inflammation related biomarkers among different age groups of pediatric patients with COVID-19 in Wuhan City, China (Fig. 1).

## Methods

### Patients

We retrospectively recruited pediatric patients confirmed of COVID-19, admitted to Wuhan Children's Hospital (the sole center for treating pediatric patients) from January 28th to April 1st in 2020. All patients were confirmed with COVID-19 by positive tests of nasopharyngeal swab specimens through real-time reverse transcription-polymerase chain reaction assay.

The severity of COVID-19 was defined based on the *Interim Guidance for Diagnosis and Treatment of Coronavirus Disease 2019* (the 6th edition) released by National Health Commission. The diagnostic criteria of different clinical stages of COVID-19 pediatric patients are listed in Supplementary Table 1. The severity of the disease was assessed on the basis of characteristics throughout the course of the disease, not just on the basis of the characteristics at admission. According to the clinical manifestation during treatment, patients were divided into five clinical stages, which are asymptomatic infection, mild, moderate, severe and critical stage.The scheme of the plan was shown in Fig. 1.

### Data collections

Demographic information, clinical characteristics (including age, sex) inflammation related and immune related laboratory results were all obtained from the Electronic Medical Record System of Wuhan Children’s Hospital. Onset symptoms and signs were also recorded. Patients could be discharged only when they met all the criteria according to the *Interim Guidance for Diagnosis and Treatment of Coronavirus Disease 2019* (the 6th edition). Days from symptom onset to admission, and average recovery time (days from admission to the day when two consecutive tests of nucleic acid confirmed negative) were both recorded.

Blood tests included: the blood routine test (white blood cell, neutrophil, lymphocyte, hemoglobin, platelet, monocyte), lymphocyte subsets analysis [proportions and cell counts of CD3+ T cell, CD3+ CD4+ T cell, CD3+ CD8+ T cell, CD19+ B cell, and natural killer (NK) cell], immune related components [immunoglobulin (Ig) G, IgM, IgA, serum complement C3 and C4], inflammation factors [interleukin (IL)-2, IL-4, IL-6, IL-10, tumor necrosis factor (TNF)-α and TNF-γ], and inflammation related markers [C-reactive protein, lactate dehydrogenase (LD), globulin and procalcitonin (PCT)]. Lymphocyte subsets analysis was conducted through flow cytometry. Immune globulin was performed through colloidal gold-based immunochromatographic strip assay. All laboratory tests were conducted on admission.

### Statistical analysis

Patients were divided into four age groups [≤ 1 year, 1–6 years (1 year < age < 7 years), 7–10 years (7 years ≤ age < 11 years), 11–15 years (11 years ≤ age < 16 years)]. Clinical characteristics including gender, severity of disease, onset symptoms and signs, and days from the onset of symptoms to discharge were described among four different age groups. All laboratory results mentioned above were defined because of their relation to our normal range (below, normal and above), and the distributions among different age groups were also displayed. Normal ranges of different blood indicators were age adjusted. Student’s *t* test for continuous variable and Chi-squared tests for categorical variables were conducted to compare the differences between four age groups. All analyses were performed by aid of Statistical Product and Service Solutions (SPSS Inc., Chicago, IL, USA) software. Distribution histograms were plotted by GraphPad PRISM software version 5 (Graph Pad Software Inc., San Diego, CA, USA, 2005). *P* value of less than 0.05 was defined as meaningful. Ethical approval was obtained by the ethical committee of Wuhan Children's Hospital.

## Results

### Demographic and clinical characteristics among pediatric patients infected with COVID-19

From January 28th to April 1st in 2020, of 241 patients with COVID-19, only 217 (90.0%) patients with moderate stage were included for analysis, excluding 10, 6, and 8 patients with mild, severe, and critical state disease, respectively. No asymptomatic cases were found among included inpatients. To avoid bias from different clinical stage, 217 (90.0%) patients that belonged to moderate stage were finally included. Clinical characteristics are summarized in Table 1. The median age is 6.9 years (range 2 months to 15 years). The majority of patients were boys (*n* = 135, 62.2%), and no significant differences were found between boys and girls. Fever (59.0%) and cough (59.9%) were the most common clinical manifestations. Other common symptoms included diarrhea (12.0%), nasal congestion (8.8%), fatigue (7.8%), vomiting (9.7%), and tachypnea (3.2%).

**Table 1:** Characteristics of children coronavirus disease 2019 cases

Clinical characteristics	All included patients (*n* = 217)	≤ 1 y patients (*n* = 40)	1–6 y patients (*n* = 58)	7–10 y patients (*n* = 65)	11–15 y patients (*n* = 54)	*P*
Age (y), median (range)	6.9 (0.17–15)	0.5 (0.17–0.83)	3.1 (1.1–5.7)	8.2 (7–10.9)	12.9 (11–15.6)	–
Gender, *n* (%)						
Boy	135 (62.2)	25 (62.5)	33 (56.9)	42 (64.6)	35 (64.8)	0.798
Girl	82 (37.8)	15 (37.5)	25 (43.1)	23 (35.4)	19 (35.2)	
Signs and symptoms, *n* (%)						
Fever	128 (59.0)	30 (75.0)	33 (56.9)	33 (50.8)	33 (61.1)	–
Cough	130 (59.9)	28 (70.0)	35 (60.3)	34 (52.3)	34 (63.0)	
Diarrhea	26 (12.0)	8 (20.0)	7 (12.1)	6 (9.2)	4 (7.4)	
Nasal congestion	19 (8.8)	5 (12.5)	5 (8.6)	4 (6.2)	4 (7.4)	
Fatigue	7 (7.8)	2 (5.0)	3 (5.2)	5 (7.7)	5 (9.3)	
Vomiting	21 (9.7)	4 (10.0)	5 (8.6)	7 (10.8)	5 (9.3)	
Tachypnea on admission	7 (3.2)	2 (5.0)	1 (1.7)	1 (1.5)	5 (9.3)	
Days from admission to two negative tests of nucleic acid, median (range)	22.26 (13–52)	25.63 (13–52)	25.56 (14–50)	22.27 (18–47)	19.53 (15–46)	0.001

“–” not available

After dividing patients into different age groups, significant differences were found in recovery time (days from the onset of symptoms to discharge) among different age groups (*P* = 0.001). The average recovery time among children of older than 6 years (7–10 years, mean 22.27 days; 11–15 years, mean 19.53 days) was significantly shorter than children younger than 6 years (≤ 1 year, mean 25.63 days; 1–6 years, mean 25.56 days). No other statistically significant differences were found among different age groups in sex, signs and symptoms.

### Blood routine results among COVID-19 pediatric patients of moderate stage

We further carried out a comparative analysis of hematological indicators of children among different age groups. Routine blood results upon admission are listed in Table 2 and Supplementary Fig. 1. As shown in Table 2, the majority of results of the confirmed cases were within reference range, such as the white blood cell (*n* = 146, 67.3%), neutrophil (*n* = 187, 86.2%), lymphocyte (*n* = 182, 83.9%), hemoglobin (*n* = 157, 72.4%), and platelet (*n* = 171, 78.8%). Among all the 217 patients, 65 (30.0%) children suffered from leukopenia. Only 14 (6.5%) patients had lymphopenia, and only 13 (6.0%) patients had elevated levels of neutrophil. However, half of the patients (*n* = 113, 52.1%) had elevated levels of monocyte, and one fifth of the patients (*n* = 45, 20.7%) suffered from thrombocytosis.

**Table 2:** Blood routine results of children with coronavirus disease 2019

Variables	All patients (*n* = 217)	< 1 y patients (*n* = 40)	1–6 y patients (*n* = 58)	7–10 y patients (*n* = 65)	11–15 y patients (*n* = 54)	*P*
White blood cell count (× 10^9^/L)	**Normal range (age adjusted)**	**6.0–15.5**	**5.0–15.5**	**4.5–13.5**	**4.5–13.0**	
Mean ± SD	6.86 ± 0.17	8.21 ± 0.50	7.47 ± 0.31	6.21 ± 0.27	5.98 ± 0.24	
Below normal, *n* (%)	65 (30.0)	7 (17.5)	10 (17.2)	27 (41.5)	21 (38.9)	0.009
Normal, *n* (%)	146 (67.3)	30 (75.0)	47 (81.0)	37 (56.9)	32 (59.3)	
Above normal, *n* (%)	6 (2.8)	3 (7.5)	1 (1.7)	1 (1.5%)	1 (1.9)	
Neutrophil count (× 10^9^/L)	**Normal range (age adjusted)**	**1.5–8.5**	**1.5–8.0**	**1.5–8.5**	**1.8–8.0**	
Mean ± SD	2.91 ± 0.11	2.39 ± 0.27	2.83 ± 0.24	3.05 ± 0.18	3.23 ± 0.18	
Below normal, *n* (%)	17 (7.8)	8 (20.0)	6 (10.3)	3 (4.6)	0 (0.0)	0.019
Normal, *n* (%)	187 (86.2)	29 (72.5)	48 (82.8)	59 (90.8)	51 (94.4)	
Above normal, *n* (%)	13 (6.0)	3 (7.5)	4 (6.9)	3 (4.6)	3 (5.6)	
Lymphocyte count (× 10^9^/L)	**Normal range (age adjusted)**	**4.0–10.5**	**2.0–8.0**	**1.5–6.5**	**1.2–5.2**	
Mean ± SD	3.24 ± 0.12	4.89 ± 0.37	3.87 ± 0.24	2.56 ± 0.13	2.16 ± 0.82	
Below normal, *n* (%)	14 (6.5)	4 (10.0)	4 (6.9)	3 (4.6)	3 (5.6)	< 0.001
Normal, *n* (%)	182 (83.9)	24 (60.0)	46 (79.3)	62 (95.4)	50 (92.6)	
Above normal, *n* (%)	21 (9.7)	12 (30.0)	8 (13.8)	0 (0.0)	1 (1.9)	
Hemoglobin (g/L)	**Normal range (age adjusted)**	**113–141**	**115–140**	**115–155**	**120–160**	
Mean ± SD	126 ± 0.92	113 ± 1.99	124 ± 1.18	127 ± 1.25	136 ± 1.82	
Below normal, *n* (%)	32 (14.7)	19 (47.5)	5 (8.6)	5 (7.7)	3 (5.6)	< 0.001
Normal, *n* (%)	157 (72.4)	21 (52.5	51 (87.9)	55 (84.6)	30 (55.6)	
Above normal, *n* (%)	28 (12.9)	0 (0.0)	2 (3.4)	5 (7.7)	21 (38.9)	
Platelet count (× 10^9^/L)	**Normal range (age adjusted)**	**150–400**	**100–378**	**100–378**	**100–378**	
Mean ± SD	305 ± 7.09	389 ± 22.81	303 ± 11.37	286 ± 10.07	268 ± 10.53	
Below normal, *n* (%)	1 (0.5)	0 (0.0)	0 (0.0)	0 (0.0)	1 (1.9)	< 0.001
Normal, *n* (%)	171 (78.8)	18 (45.0)	45 (77.6)	58 (89.2)	50 (92.6)	
Above normal, *n* (%)	45 (20.7)	22 (55.0)	13 (22.4)	7 (10.8)	3 (5.6)	
Monocyte (× 10^9^ g/L)	**Normal range (age adjusted)**	**0.26–1.7**	**0.26–0.4**	**0.26–0.4**	**0.26–0.4**	
Mean ± SD	0.47 ± 0.01	0.56 ± 0.42	0.48 ± 0.03	0.41 ± 0.02	0.44 ± 0.02	
Below normal, *n* (%)	21 (9.7)	1 (2.5)	2 (3.4)	11 (16.9)	7 (13.0)	0.022
Normal, *n* (%)	83 (38.2)	11 (27.5)	28 (48.3)	25 (38.5)	19 (35.2)	
Above normal, *n* (%)	113 (52.1)	28 (70.0)	28 (48.3)	29 (44.6)	28 (51.9)	

Normal range (age-adjusted) of each item has been shown in bold. *SD* standard deviation

With the increase of children's age, patients with reduced levels of white blood cell significantly increased (≤ 1 year, *n* = 7, 17.5%; 1–6 years, *n* = 10, 17.2%; 7–10 years, *n* = 27, 41.5%; 11–15 years, *n* = 21, 38.9%; *P* = 0.009). Similarly, patients were also more unlikely to have reduced neutrophil with the decrease of age (11–15 years, *n* = 0, 0%; 7–10 years, *n* = 3, 4.6%; 1–6 years, *n* = 6, 10.3%; ≤ 1 year, *n* = 8, 20.0%; *P* = 0.019). Increased neutrophils and decreased lymphocytes, which correlate significantly with the disease severity and death, were found to have no difference between different age groups. However, elevated lymphocyte (*P* < 0.001), anemia (*P* < 0.001), and thrombocytosis (*P* < 0.001) were significantly found in patients under 1 year old. Specifically speaking, increased levels of lymphocyte were mostly seen among children under 1 year old (≤ 1 year, *n* = 12, 30%; 1–6 years, *n* = 8, 13.8%; 7–10 years, *n* = 0, 0%; 11–15 years, *n* = 1, 1.9%). Moreover, children under 1 year old were more vulnerable to suffer from anemia (≤ 1 year, *n* = 10, 47.5%; 1–6 years, *n* = 5, 8.6%; 7–10 years, *n* = 5, 7.7%; 11–15 years, *n* = 3, 5.6%; *P* < 0.001) and thrombocythemia (≤ 1 year, *n* = 22, 55.0%; 1–6 years, *n* = 13, 22.4%; 7–10 years, *n* = 7, 10.8%; 11–15 years, *n* = 3, 5.6%, *P* < 0.001). Levels of monocyte were also most elevated among patients under 1 year old (≤ 1 year, *n* = 28, 70%; 1–6 years, *n* = 28, 48.3%; 7–10 years, *n* = 29, 44.6%; 11–15 years, *n* = 28, 51.9%; *P* = 0.022).

### Lymphocyte subsets, immune related components, inflammatory cytokines, inflammatory related biomarkers in pediatric patients

Immune and inflammation related indicators were also analyzed and listed in Table 3 and Supplementary Figs. 2–4. From the analysis of lymphocyte subsets (Table 3 and Sup. Figure 2), the majority of the results of CD3+ T cell count (*n* = 184, 84.8%), CD3+ CD4+ T cell count (*n* = 175, 80.6%) and CD3+ CD8+ T cell count (*n* = 191, 88.0%) were within reference range. No significant differences were found in the proportion of CD3+ T cell and CD3+ CD4+ T cell. For CD3+ CD8+ T cell, there are also no significant differences among four age groups on CD3+ CD8+ T cell count and proportion. Notably for B cells, elevated proportions of CD19+ B cell were mostly seen in patients under 1 year (*n* = 12, 30.0%, *P* < 0.001). Correspondingly, elevated levels of CD19+ B cell count were most found among patients less than 6 years old, especially among children under 1 year old (≤ 1 year, *n* = 13, 32.5%; 1–6 years, *n* = 9, 15.5%; 7–10 years, *n* = 1, 1.5%; 11–15 years, *n* = 0, 0%; *P* < 0.001). Decreased NK cell proportions were mostly found among patients under 10 years old (≤ 1 year, *n* = 13, 32.5%; 1–6 years, *n* = 21, 36.2%; 7–10 years, *n* = 25, 38.5%; 11–15 years, *n* = 7, 13.0%; *P* = 0.033).

**Table 3:** Results of lymphocyte subpopulation and inflammatory factor children with coronavirus disease 2019

Variables	All patients (*n* = 217)	< 1 y patients (*n* = 40)	1–6 y patients (*n* = 58)	7–10 y patients (*n* = 65)	11–15 y patients (*n* = 54)	*P*
Lymphocyte subpopulation						
CD3+ T, %	**Normal range**	**38.56–70.06**	**38.56–70.06**	**38.56–70.06**	**38.56–70.06**	
< 38.56, *n* (%)	0 (0.0)	0 (0.0)	0 (0.0)	0 (0.0)	0 (0.0)	0.347
38.56–70.06, *n* (%)	125 (57.6)	28 (70.0)	36 (62.1)	31 (47.7)	30 (55.6)	
> 70.06, *n* (%)	84 (38.7)	11 (27.5)	19 (32.8)	32 (49.2)	22 (40.7)	
CD3+ T count (/µL)	**Normal range (age adjusted)**	**1400–6700**	**900–4500**	**700–4200**	**800–3500**	
< 805, *n* (%)	3 (1.4)	0 (0.0)	0 (0.0)	1 (1.5)	2 (3.7)	0.001
805–4459, *n* (%)	184 (84.8)	26 (65.0)	49 (84.5)	59 (90.8)	50 (92.6)	
> 4459, *n* (%)	16 (7.4)	9 (22.5)	6 (10.3)	1 (1.5)	0 (0)	
CD3+ CD4+ T, %	**Normal range**	**14.21–36.99**	**14.21–36.99**	**14.21–36.99**	**14.21–36.99**	
< 14.21, *n* (%)	4 (1.8)	0 (0.0)	1 (1.7)	2 (3.1)	1 (1.9)	0.581
14.21–36.99, *n* (%)	124 (57.1)	19 (47.5)	31 (53.4)	37 (56.9)	37 (68.5)	
> 36.99, *n* (%)	81 (37.3)	20 (50.0)	23 (39.7)	24 (36.9)	14 (25.9)	
CD3+ CD4+ T (/µL)	**Normal range (age adjusted)**	**1000–4600**	**500–2400**	**300–2000**	**400–2100**	
< 345, *n* (%)	8 (3.7)	2 (5.0)	0 (0.0)	3 (4.6)	3 (5.6)	< 0.001
345–2350, *n* (%)	175 (80.6)	21 (52.5)	47 (81.0)	58 (89.2)	49 (90.7)	
> 2350, *n* (%)	20 (9.2)	12 (30.0)	8 (13.8)	0 (0.0)	0 (0)	
CD3+ CD8+ T, %	**Normal range**	**13.24–38.53**	**13.24–38.53**	**13.24–38.53**	**13.24–38.53**	
< 13.24, *n* (%)	4 (1.8)	3 (7.5)	0 (0.0)	1 (1.5)	0 (0.0)	0.095
13.24–38.53, *n* (%)	195 (89.9)	36 (90.0)	54 (93.1)	57 (87.7)	48 (88.9)	
> 38.53, *n* (%)	10 (4.6)	0 (0.0)	1 (1.7)	5 (7.7)	4 (7.4)	
CD3+ CD8+ T (/µL)	**Normal range (age adjusted)**	**400–2100**	**300–1600**	**300–1800**	**200–1200**	
< 314, *n* (%)	3 (1.4)	0 (0.0)	0 (0.0)	2 (3.1)	1 (1.9)	0.337
314–2080, *n* (%)	191 (88.0)	32 (80.0)	51 (87.9)	58 (89.2)	50 (92.6)	
> 2080, *n* (%)	9 (4.1)	3 (7.5)	4 (6.9)	1 (1.5)	1 (1.9)	
CD19+ B, %	**Normal range**	**10.86–28.03**	**10.86–28.03**	**10.86–28.03**	**10.86–28.03**	
< 10.86, *n* (%)	12 (5.5)	2 (5.0)	3 (5.2)	3 (4.6)	4 (7.4)	< 0.001
10.86–28.03, *n* (%)	180 (82.9)	25 (62.5)	49 (84.5)	60 (92.3)	46 (85.2)	
> 28.03, *n* (%)	17 (7.8)	12 (30.0)	3 (5.2)	0 (0.0)	2 (3.7)	
CD19+ B (/µL)	**Normal range (age adjusted)**	**600–2700**	**200–2100**	**200–1600**	**200–600**	
< 240, *n* (%)	13 (6.0)	0 (0.0)	3 (5.2)	5 (7.7)	3 (5.2)	< 0.001
240–1317, *n* (%)	167 (77.0)	22 (55.0)	43 (74.1)	55 (84.6)	43 (74.1)	
> 1317, *n* (%)	23 (10.6)	13 (32.5)	9 (15.5)	1 (1.5)	9 (15.5)	
NK cell, %	**Normal range**	**7.92–33.99**	**7.92–33.99**	**7.92–33.99**	**7.92–33.99**	
< 7.92, *n* (%)	66 (30.4)	13 (32.5)	21 (36.2)	25 (38.5)	7 (13.0)	0.033
7.92–33.99, *n* (%)	130 (59.9)	23 (57.5)	30 (51.7)	33 (50.8)	44 (81.5)	
> 33.99, *n* (%)	5 (2.3)	0 (0.0)	3 (5.2)	1 (1.5)	1 (1.9)	
NK cell (/µL)	**Normal range (age adjusted)**	**230–801**	**155–724**	**120–483**	**87–504**	
< 210, *n* (%)	55 (25.3)	8 (20.0)	19 (32.8)	20 (30.8)	8 (14.8)	0.250
210–1514, *n* (%)	144 (66.4)	27 (67.5)	35 (60.3)	39 (60.0)	43 (79.6)	
> 1514, *n* (%)	2 (0.9)	1 (2.5)	0 (0.0)	0 (0.0)	1 (1.9)	
Total missing data for each item in lymphocyte subpopulation	8 (3.7)	1 (2.5)	3 (5.2)	2 (3.1)	2 (3.7)	
Immune related factors						
IgG (g/L), *n* (%)	**Normal range (age adjusted)**	**3.45–12.13**	**4.41–11.35**	**6.33–12.80**	**6.39–13.49**	
Missing data	16 (7.4)	3 (7.5)	6 (10.3)	7 (10.8)	0 (0.0)	
< 3.58	13 (6.0)	11 (27.5)	2 (3.4)	0 (0.0)	0 (0.0)	< 0.001
3.58–10.69	122 (56.2)	25 (62.5)	39 (67.2)	33 (50.8)	25 (46.3)	
> 10.69	66 (30.4)	1 (2.5)	11 (19.0)	25 (38.5)	29 (53.7)	
IgM (g/L), *n* (%)	**Normal range (age adjusted)**	**0.21–0.86**	**0.23–1.00**	**0.24–1.21**	**0.28–1.76**	
Missing data	16 (7.4)	3 (7.5)	6 (10.3)	7 (10.8)	0 (0.0)	
< 0.11	0 (0.0)	0 (0.0)	0 (0.0)	0 (0.0)	0 (0.0)	< 0.001
0.11–1.06	129 (59.4)	35 (87.5)	31 (53.4)	28 (43.1)	35 (64.8)	
> 1.06	72 (33.2)	2 (5.0)	21 (36.2)	30 (46.2)	19 (35.2)	
IgA (g/L), *n* (%)	**Normal range (age adjusted)**	**0.07–0.53**	**0.11–0.80**	**0.22–1.18**	**0.35–1.56**	
Missing data	16 (7.4)	3 (7.5)	6 (10.3)	7 (10.8)	0 (0.0)	
< 0.22	17 (7.8)	11 (27.5)	5 (8.6)	1 (1.5)	0 (0.0)	< 0.001
0.33–1.26	69 (31.8)	22 (55.0)	36 (62.1)	5 (7.7)	6 (11.1)	
> 1.26	115 (53.0)	4 (10.0)	11 (19.0)	52 (80.0)	48 (88.9)	
Serum complement C3 (g/L), *n* (%)	**Normal range (age adjusted)**	**0.86–1.74**	**0.77–1.71**	**0.89–1.95**	**0.83–1.77**	
Missing data	13 (6.0)	3 (7.5)	2 (3.4)	8 (12.3)	0 (0.0)	
< 0.7	16 (7.4)	5 (12.5)	6 (10.3)	3 (4.6)	2 (3.7)	0.003
0.7–1.12	146 (67.3)	28 (70.0)	43 (74.1)	43 (66.2)	32 (59.3)	
> 1.12	42 (19.4)	4 (10.0)	7 (12.1)	11 (16.9)	20 (37.0)	
Serum complement C4 (g/L), *n* (%)	**Normal range (age adjusted)**	**0.12–0.39**	**0.97–0.36**	**0.12–0.32**	**0.15–0.45**	
Missing data	13 (6.0)	3 (7.5)	2 (3.4)	8 (12.3)	0 (0.0)	
< 0.1	15 (6.9)	2 (5.0)	7 (12.1)	3 (4.6)	3 (5.6)	0.091
0.1–0.38	179 (82.5)	34 (85.0)	47 (81.0)	52 (80.0)	46 (85.2)	
> 0.38	10 (4.6)	1 (2.5)	2 (3.4)	2 (3.1)	5 (9.3)	
Inflammatory factors						
IL-2 (pg/mL), *n* (%)	**Normal range**	**0–14**	**0–14**	**0–14**	**0–14**	
Missing data	7 (3.2)	1 (2.5)	3 (5.2)	3 (4.6)	0 (0.0)	
0–14	209 (96.3)	39 (97.5)	55 (94.8)	61 (93.8)	54 (100)	0.499
> 14	1 (0.5)	0 (0.0)	0 (0.0)	1 (1.5)	0 (0.0)	
IL-4 (pg/mL), *n* (%)	**Normal range**	**0–12.9**	**0–12.9**	**0–12.9**	**0–12.9**	
Missing data	7 (3.2)	1 (2.5)	3 (5.2)	3 (4.6)	0 (0.0)	
0–12.9	206 (94.9)	36 (90.0)	55 (94.8)	61 (93.8)	54 (100)	0.058
> 12.9	4 (1.8)	3 (7.5)	0 (0.0)	1 (1.5)	0 (0.0)	
IL-6 (pg/mL), *n* (%)	**Normal range**	**0–20.9**	**0–20.9**	**0–20.9**	**0–20.9**	
Missing data	7 (3.2)	1 (2.5)	3 (5.2)	3 (4.6)	0 (0.0)	
0–20.9	172 (79.3)	26 (65.0)	43 (74.1)	52 (80.0)	51 (94.4)	0.015
> 20.9	38 (17.5)	13 (32.5)	12 (20.7)	10 (15.4)	3 (5.6)	
IL-10 (pg/mL), *n* (%)	**Normal range**	**0–5.9**	**0–5.9**	**0–5.9**	**0–5.9**	
Missing data	7 (3.2)	1 (2.5)	3 (5.2)	3 (4.6)	0 (0.0)	
0–5.9	155 (71.4)	22 (55.0)	37 (63.8)	50 (76.9)	46 (85.2)	0.017
> 5.9	55 (25.3)	17 (42.5)	18 (31.0)	12 (18.5)	8 (14.8)	
TNF-α (pg/mL), *n* (%)	**Normal range**	**0–5.5**	**0–5.5**	**0–5.5**	**0–5.5**	
Missing data	7 (3.2)	1 (2.5)	3 (5.2)	3 (4.6)	0 (0.0)	
0–5.5	178 (82.0)	30 (75.0)	47 (81.0)	51 (78.5)	50 (92.6)	0.252
> 5.5	32 (14.7)	9 (22.5)	8 (13.8)	11 (16.9)	4 (7.4)	
TNF-γ (pg/mL), *n* (%)	**Normal range**	**0–17.3**	**0–17.3**	**0–17.3**	**0–17.3**	
Missing data	7 (3.2)	1 (2.5)	3 (5.2)	3 (4.6)	0 (0.0)	
0–17.3	202 (93.1)	34 (85.0)	53 (91.4)	61 (93.8)	54 (100)	0.023
> 17.3	8 (3.7)	5 (12.5)	2 (3.4)	1 (1.5)	0 (0.0)	
Inflammatory related markers						
CRP (mg/L), *n* (%)	**Normal range**	**0–3**	**0–3**	**0–3**	**0–3**	
Missing data	28 (12.9)	6 (15.0)	7 (12.1)	9 (13.8)	6 (11.1)	
0–3	149 (68.7)	24 (60.0)	43 (74.1)	46 (70.8)	36 (66.7)	0.748
> 3	40 (18.4)	10 (25.0)	8 (13.8)	10 (15.4)	12 (22.2)	
LD (U/L), *n* (%)	**Normal range**	**120–300**	**120–300**	**120–300**	**120–300**	
Missing data	10 (4.6)	2 (5.0)	2 (3.4)	4 (6.2)	2 (3.7)	
< 120	1 (0.5)	0 (0.0)	1 (1.7)	0 (0.0)	0 (0)	0.002
120–300	156 (71.9)	20 (50.0)	37 (63.8)	51 (78.5)	48 (88.9)	
> 300	50 (23.0)	18 (45.0)	18 (31.0)	10 (15.4)	4 (7.4)	
Globulin (g/L), *n* (%)	**Normal range**	**20–40**	**20–40**	**20–40**	**20–40**	
Missing data	11 (5.1)	5 (12.5)	2 (3.4)	1 (1.5)	3 (5.6)	
< 20	51 (23.5)	17 (42.5)	23 (39.7)	7 (10.8)	4 (7.4)	< 0.001
20–40	153 (70.5)	17 (42.5)	33 (56.9)	57 (87.7)	46 (85.2)	
> 40	2 (0.9)	1 (2.5)	0 (0.0)	0 (0.0)	1 (1.9)	
PCT (ng/mL), *n* (%)	**Normal range**	**0–0.05**	**0–0.05**	**0–0.05**	**0–0.05**	
Missing data	10 (4.6)	1 (2.5)	4 (6.9)	3 (4.6)	2 (3.7)	
0–0.05	107 (49.3)	11 (27.5)	30 (51.7)	32 (49.2)	34 (63.0)	0.027
> 0.05	100 (46.1)	28 (70.0)	24 (41.4)	30 (46.2)	18 (33.3)	

Normal range (age-adjusted) of each item has been shown in bold. *NK* natural killer, *Ig* immunoglobulin, *IL* interleukin, *TNF* tumor necrosis factor, *CRP* C-reactive protein, *LD* lactate dehydrogenase, *PCT* procalcitonin

From the results of immune related components analysis (Table 3 and Supplementary Fig. 3), low levels of IgG and IgA were mostly found among patients less than 1 year (IgG: *n* = 11, 27.5%, *P* < 0.001; IgA: *n* = 11, 27.5%, *P* < 0.001). Elevated IgA were mostly found among patients more than 7 years old (≤ 1 year, *n* = 4, 10.0%; 1–6 years, *n* = 11, 19.0%; 7–10 years, *n* = 52, 80.0%; 11–15 years, *n* = 48, 88.9%; *P* < 0.001). Moreover, with the increase of children's age, more patients with elevated levels of IgA were found. Patients with elevated levels of IgM were the least among children under 1 year old (*n* = 2, 5.0%, *P* < 0.001). For serum complement C4, there existed no significant differences among different age groups (*P* = 0.091). However, high levels of serum complement C3c were mostly seen in children 11–15 years old (≤ 1 year, *n* = 4, 10.0%; 1–6 years, *n* = 7, 12.1%; 7–10 years, *n* = 11, 16.9%; 11–15 years, *n* = 20, 37%), whereas low levels of serum complement C3c were significantly most observed in children younger than 1 year old (≤ 1 year, *n* = 5, 12.5%; 1–6 years, *n* = 6, 10.3%; 7–10 years, *n* = 3, 4.6%; 11–15 years, *n* = 2, 3.7%; *P* = 0.003).

From the results of inflammatory cytokines and related biomarkers (Table 3 and Supplementary Fig. 4), compared with children of 11–15 years old, elevated levels of IL-6 were mostly found in children under 11 years old (< 11 years, *n* = 35; 11–15 years, *n* = 3; *P* = 0.015), so as the elevated levels of IL-10 (< 11 years, *n* = 47; 11–15 years, *n* = 8; *P* = 0.017), TNF-γ (< 11 years, *n* = 8; 11–15 years, *n* = 0; *P* = 0.023) and LD (< 11 years, *n* = 46; 11–15 years, *n* = 4; *P* = 0.002). Patients under 1 year old more intended to have elevated levels of PCT (≤ 1 year, *n* = 28, 70.0%; 1–6 years, *n* = 24, 41.4%; 7–10 years, *n* = 30, 46.2%; 11–15 years, *n* = 18, 33.3%; *P* = 0.027).

## Discussion

As the world epidemic of COVID-19, people of all ages are susceptible to SARS-CoV-2 infection. Previous reports have focused mainly on adult patients. However, data among pediatric patients were still limited. As we all know, children of different ages may differ in immune status. With the increase of age, both the innate and acquired immunity gradually mature [9]. Thus far, there are only three retrospective clinical studies among Chinese pediatric patients [4, 6, 10]. However, none of them compared the immune and inflammation levels among different ages of pediatric patients. Others were all in quite small-scale, such as case reports [7, 11]. To the best of our knowledge, we are the first to retrospectively compare the immune and inflammation status among different age groups of children from Wuhan, China.

From our results, the majority of the patients (*n* = 217, 90.0%) had only moderate symptoms, which coincides with other studies involving pediatric patients [4, 7]. Those patients ranged from 2 months to 15 years. Most of them were boys (61.1%), indicating that boys seemed to be more susceptible to SARS-CoV-2 infection. Those were similar to the results of Dong et al.’s study [4], which 57.5% (420 out of 731) of the pediatric patients were boys. Our study also for the first time indicated that children under 1 year old are much more likely to suffer from anemia and thrombocythaemia. In addition, increased neutrophils and decreased lymphocytes correlate significantly with disease severity and death [10]. In one report of 41 cases among adults in Wuhan, increased neutrophils and reduced lymphocytes were observed to be statistically different among patients of intensive care unit (ICU) vs. non-ICU care [12]. In our results, all included patients had moderate symptoms, and no differences were found in increased neutrophils and in decreased lymphocytes between different age groups. According to genomic sequence comparison, SARS-CoV-2 shares most of the genomic similarity with Middle East respiratory syndrome (MERS)-CoV and with SARS-CoV, approximately 50% and 79%, respectively [13]. Similarly, in severe or lethal cases of other SARS-CoV or MERS-CoV infection, increased neutrophil and decreased lymphocyte are also consistently observed [14, 15]. In our study the majority of the results of white blood cell (*n* = 146, 67.3%), neutrophil (*n* = 187, 86.2%), and lymphocyte (*n* = 182, 83.9%) among pediatric patients are within reference range, which may coincide with children's mild symptoms.

In terms of humoral immunity, patients with elevated B cell count were mostly seen among children under 1 year old, so as the number of patients with elevated proportions of B cells. These results suggested that B cells may play an important role in the immune response of COVID-19 among patients under 1 year old. However, early protection against many infectious diseases is mostly given by the passive IgG antibody transferred from the mother in milk [16, 17]. Usually, IgG remains a low level until 2 years old, so as to IgM and IgA, which is as the same shown in our reports. Moreover, patients with elevated levels of C3c were only found in older children. These results suggested that it may be more difficult for children under 1 year old to eliminate the virus through the activation of sufficient immune responses; thus, high severity rate and the longer hospitalization time among children under 1 year old may happen.

NK cells play an important role in restraining viral replication and dissemination before adaptive immunity is established [18]. The cytolytic function of NK cell is only half of adult level at birth. In our study younger patients (< 11 years) tended to have decreased proportions of NK cell, which indicated that NK cell may be impaired in fighting against SARS-CoV-2 among younger patients. In one study of 128 convalescent patients with SARS-CoV infection, effective T cell responses were shown to be significantly associated with higher neutralizing antibody and with more serum Th2 cytokines (IL-4 and IL-10) detected in fatal group [19]. Our research showed that there existed no significant differences in either proportion or cell count of CD3+ CD8+ T cell, CD3+ CD4+ T cell and CD3+ T cell among different age groups of patients. Only elevated levels of CD19+ B cell count were most found among patients less than 6 years old. Hence, the inadequate production of antibodies and impaired effect of functional T cell may reflect the unique immune system among children fighting against COVID-19, especially patients younger than 1 year old.

Cytokines and chemokines have been assumed to play an important role in immunity and immunopathology during virus infections [20, 21]. Studies have demonstrated that pro-inflammatory cytokines and chemokines are predictive of severe clinical outcomes among adult COVID-19 patients [22, 23]. Excessive inflammation may increase organ damage. In our study patients younger than 1 year old had a stronger inflammatory response, with the evidence of more patients with elevated levels of IL-6, IL-10, and TNF-γ. In addition, the levels of inflammatory markers, such as LD and PCT, also increased more significantly in the patients younger than 1 year old. While, among patients of 11–15 years, they have the lowest number of patients with elevated levels of TNF-α, though there is no significance among different groups (< 11 years, *n* = 28; 11–15 years, *n* = 4; *P* = 0.252). Therefore, the inflammatory response may be more intense in patients under 1 year old.

In Sun et al.'s study [7], three of the eight children in severe ICUs were younger than one year old. Similarly, in Dong et al.'s study [4], under different ages, children younger than 1 year old had a more severe rate of 53.8%. In our study, owing to the incomplete development of cellular and humoral immunity together with much more intense inflammatory reaction, these results may explain why children under 1 year old are much more likely to progress into severe cases. This may also be one of the potential reasons why the course of disease among patients under 1 year old is much longer than that of other age groups. This research may provide valuable clues for the prevention, treatment, and mechanism of COVID-19 among different ages of pediatric patients.

Our study also has limitations. First, the blood results from our study were measured only once on admission. The subsequent changes of the immune and inflammatory status among different age groups of COVID-19 pediatric patients are still needed to be dynamically tracked and analyzed. Second, we conducted this study in one-centered and retrospective way. Larger samples of studies are still needed to verify our conclusions in the future.

In conclusion, patients under 1 year old suffered from stronger inflammatory response (IL-6, IL-10, TNF-γ) and immune responses (CD19+ B cell, reduced neutrophils and increased lymphocytes). However, owing to the insufficient production of antibodies and serum complements, all may contribute to inadequate immune reactions in eliminating virus, which should be paid more attention.

## Supplementary Information

Below is the link to the electronic supplementary material.

Supplementary file1 (DOCX 15 KB)

Supplementary file2 (TIF 3279 KB)

Supplementary file3 (TIF 2340 KB)

Supplementary file4 (TIF 2296 KB)

Supplementary file5 (TIF 3579 KB)

Supplementary file6 (DOCX 14 KB)
