# Supplementary material for: Characteristics of immune and inflammatory responses among different age groups of pediatric patients with COVID-19 in China
Source: World J Pediatr. 2021 Aug 2;17(4):375–84. doi: 10.1007/s12519-021-00440-1 (PMC8328122; doi:10.1007/s12519-021-00440-1)
Supplement: Supplementary file 1 — Supplementary file1 (DOCX 15 KB) [file 12519_2021_440_MOESM1_ESM.docx]

**Supplementary Fig. 1** Lymphocyte subpopulation analysis showed in box plots, which includes CD3+ T cell count (**a**), CD3+CD4+ T cell count (**b**), CD3+CD8+ T cell count (**c**), NK cell count (**d**), Th/Ts (**e**), CD19+ B cell count (**f**), and monocyte cell count (**g**). Each group was compared with patients under 1 year old through Student’s *t* test analysis. Gray dotted band represents normal range. *NK* natural killer, *Th* helper T cells, *Ts* suppressor T cells. ^*^*P* < 0.05, ^†^*P* < 0.01, ^‡^*P* < 0.001 vs. under 1 year group**Supplementary Fig. 2** Routine blood test analysis showed in box plots, which includes white blood cell count (**a**), neutrophil count (**b**), lymphocyte count (**c**), Hb (**d**), and platelet count (**e**). Each group was compared with patients under 1year old through Student’s *t* test analysis. Gray dotted band represents normal range. *Hb* hemoglobin. ^*^*P* < 0.05, ^‡^*P* < 0.001 vs. under 1 year group

**Supplementary Fig. 3** Routine blood test analysis showed in box plots, which includes IgG (**a**), IgM (**b**), IgA (**c**), serum complement C3 (**d**), and serum complement C4 (**e**). Each group was compared with patients under 1 year old through Student’s *t* test analysis. Gray dotted band represents normal range. *Ig* immunoglobulin. ^‡^*P* < 0.001 vs. under 1 year group

**Supplementary Fig. 4** Inflammatory factors showed in box plots, which include IL-2 (**a**), IL-4 (**b**), IL-6 (**c**), IL-10 (**d**), TNF-α (**e**), TNF-γ (**f**), LD (**g**), globulin (**h**), PCT (**i**). Each group was compared with patients under 1 year old through Student’s *t* test analysis. Gray dotted band represents normal range. *IL* interleukin, *TNF* tumor necrosis factor, *LD* lactate dehydrogenase, *PCT* procalcitonin. ^*^*P* < 0.05, ^†^*P* < 0.01, ^‡^*P* < 0.001 vs. under 1 year group
