# Supplementary figures and images for: Characteristics of immune and inflammatory responses among different age groups of pediatric patients with COVID-19 in China
Source: World J Pediatr. 2021 Aug 2;17(4):375–84. doi: 10.1007/s12519-021-00440-1 (PMC8328122; doi:10.1007/s12519-021-00440-1)

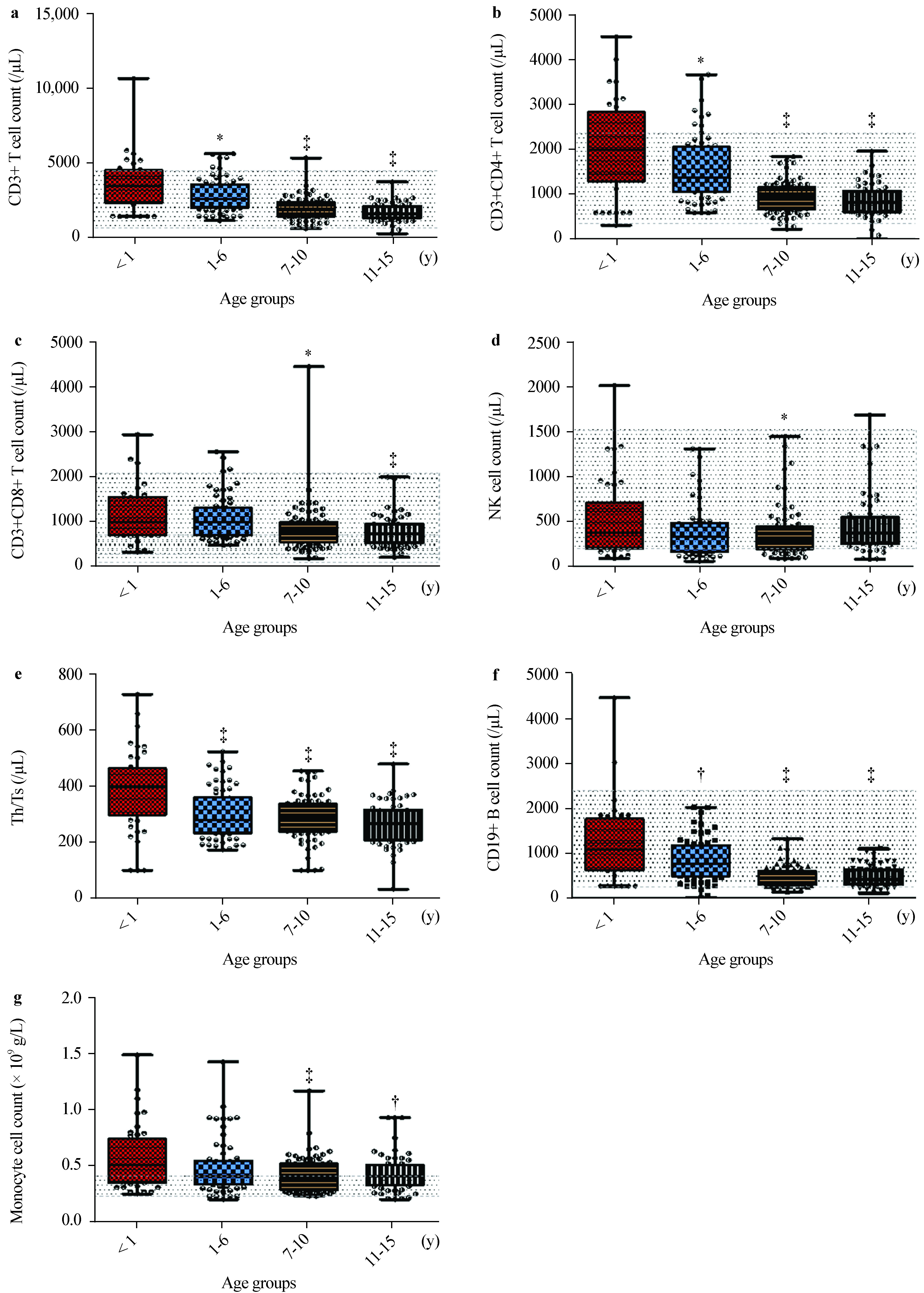

Supplement: Supplementary file 2 — Supplementary file2 (TIF 3279 KB) [file 12519_2021_440_MOESM2_ESM.tif]

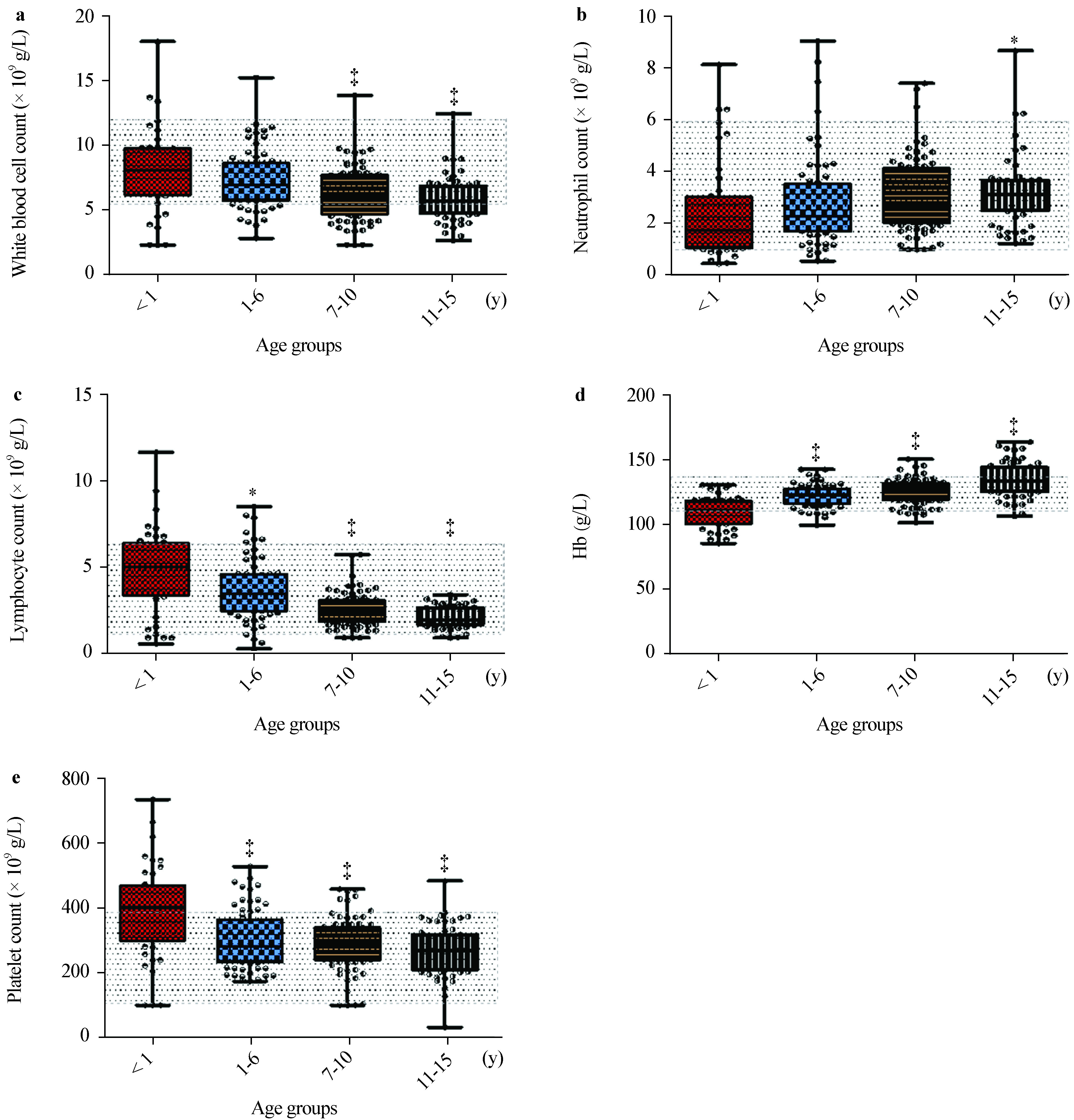

Supplement: Supplementary file 3 — Supplementary file3 (TIF 2340 KB) [file 12519_2021_440_MOESM3_ESM.tif]

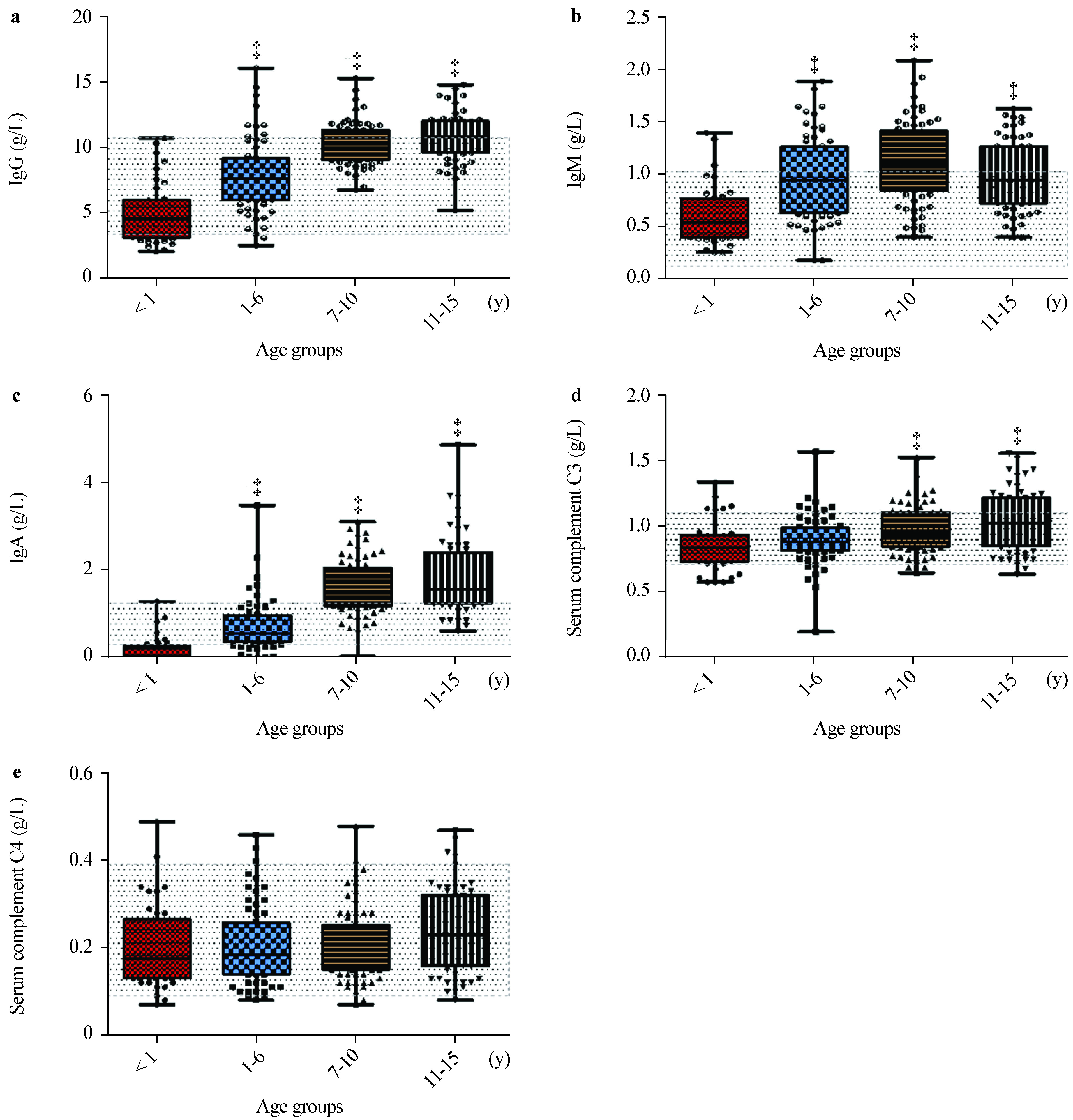

Supplement: Supplementary file 4 — Supplementary file4 (TIF 2296 KB) [file 12519_2021_440_MOESM4_ESM.tif]

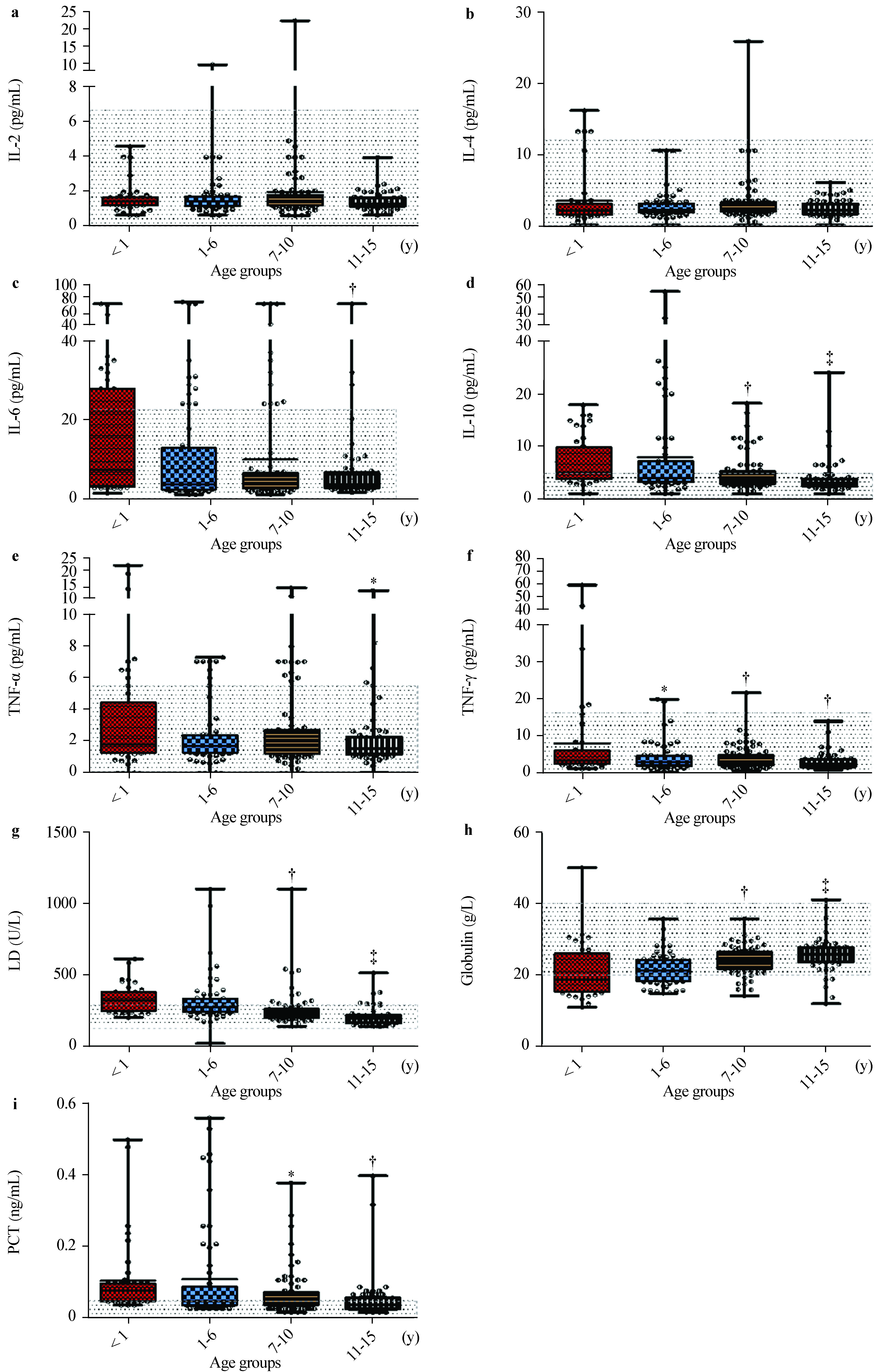

Supplement: Supplementary file 5 — Supplementary file5 (TIF 3579 KB) [file 12519_2021_440_MOESM5_ESM.tif]
