# Supplementary material for: Characteristics of immune and inflammatory responses among different age groups of pediatric patients with COVID-19 in China
Source: World J Pediatr. 2021 Aug 2;17(4):375–84. doi: 10.1007/s12519-021-00440-1 (PMC8328122; doi:10.1007/s12519-021-00440-1)
Supplement: Supplementary file 6 — Supplementary file6 (DOCX 14 KB) [file 12519_2021_440_MOESM6_ESM.docx]

| **Supplementary Table 1**. Diagnose criteria of different clinical staging of pediatric COVID-19 cases | | | | | |
| --- | --- | --- | --- | --- | --- |
| Clinical staging | Positive test of SARS-CoV-2 nucleic acid | Clinical symptoms and signs | Chest imaging of viral pneumonia | Tachypnea (≥ 30 times/min) or anoxia (SpO_2_ < 93% or FiO_2_ ≤ 300 mmHg) | Respiratory or other organ failure, shock that needs ICU care |
| Asymptomatic infection | + | - | - | - | - |
| Mild | + | + | - | - | - |
| Moderate | + | + | + | - | - |
| Severe | + | + | + | + | - |
| Critical | + | + | + | + | + |

*COVID-19* coronavirus disease 2019, *SARS-CoV-2* severe acute respiratory syndrome coronavirus 2, *SpO_2_* peripheral blood oxygen saturation, *FiO_2_* fraction of inspired oxygen, *ICU* intensive care unit. “+” positive, “-” negative
